# Correlation between Traditional Chinese Medicine Symptom Patterns and the Renal Function, Immunologic Function Index, and Blood Coagulation Index in Patients with Henoch-Schönlein Purpura Nephritis

**DOI:** 10.1155/2018/1972527

**Published:** 2018-04-17

**Authors:** Wanyu Dang, Huimin Yan, Xiaoming Wu, Xiaofang Zhen, Linyi Hou

**Affiliations:** Department of Traditional Chinese Medicine, Beijing Children's Hospital, Capital Medical University, National Center for Children's Health, Beijing 100045, China

## Abstract

**Objective:**

We investigate the correlation between the patterns of traditional Chinese medicine (TCM) syndromes and the damage of renal function, immunologic function index, and blood coagulation index in patients with Henoch-Schönlein purpura nephritis (HSPN) and thus provide the therapeutic effects of Chinese herbs decoction on HSPN.

**Methods:**

We studied 262 hospitalized patients diagnosed with HSPN between 1 February 2016 and 1 January 2017. Indexes like renal function, immunologic function, and blood coagulation were measured. The patients were classified into four different patterns of TCM symptoms.

**Results:**

In a total of 262 patients with HSPN, dampness-heat accumulation accounted for 59.5%, which is the highest proportion of TCM symptom patterns, liver-kidney yin deficiency accounted for 17.6%, qi and yin deficiency ratio reached 12.6%, and blood-heat bleeding accounted for 9.9%. 24-hour proteinuria was heavier in the dampness-heat accumulation patients who had immune disorders and were in hypercoagulative state and hyperfibrinolysis conditions.

**Conclusion:**

We analyzed and summarized the clinical characteristics of patients with HSPN and found that dampness-heat accumulation was dominant in patients and was always accompanied by immune disorders and coagulation disorders. These results provided the largest therapeutic effects of Chinese herbs decoction for clinical treatment.

## 1. Background

Henoch-Schönlein purpura nephritis is a systemic disorder characterized by leukocytoclastic vasculitis involving the capillaries and the deposition of IgA immune complexes. Renal involvement is the principal cause of morbidity and mortality in children with HSP [1, 2]. Approximately 40% of children with HSP develop nephritis, usually within 4 to 6 weeks after the initial onset of the typical purpuric rashes. Although the pathogenetic mechanisms are still not fully delineated, several studies suggested that galactose-deficient IgA1 (Gd-IgA1) is recognized by anti-glycan antibodies, leading to the formation of the circulating immune complexes and their mesangial deposition which induce renal injury in HSPN [3–6]. As to the pathogenesis, several studies suggested that the severity of renal involvement is the major factor determining the long-term outcome of children with HSPN.

Aggressive therapies for the treatment of severe HSPN, including multiple drug combination therapy and plasmapheresis, have been shown to be effective in ameliorating proteinuria and histological severity. There is no clear consensus as to which patients with Henoch-Schönlein purpura nephritis (HSPN) at risk of a poor outcome should be treated and what therapeutic regimen should be used [2, 5].

The traditional Chinese medicine (TCM) patterns of symptoms can reflect the nature of the interaction between the disease and the environment in a certain stage; they include the corresponding symptoms, signs, and tongue and pulse qualities and can reveal the etiology, disease character, disease location, and disease trend [7, 8]. Because of the flexibility and individualization of TCM syndrome differentiation, many doctors have different understandings for the syndrome differentiation and treatment of HSPN. Although the disease has a variety of patterns of TCM syndromes, the research showed that the common TCM syndrome of HSPN mainly includes four patterns: dampness-heat accumulation, liver-kidney yin deficiency, qi and yin deficiency, and purpura with syndrome of blood-heat bleeding [8, 9]. There are few reports on the relationship between TCM syndrome and renal function, immunologic function index, and blood coagulation index. This study aims to investigate the correlation between the patterns of TCM syndromes and the damage of renal function in patients with HSPN and to provide the largest therapeutic effects of Chinese herbs decoction on patients with HSPN.

## 2. Materials and Methods

### 2.1. Study Participants

The study was approved by the institutional review board, and each patient gave written informed consent.

### 2.2. Diagnostic Criteria

Each patient's western medicine diagnosis was based on the newest diagnostic standards described in “Zhu Futang Practice of Pediatrics (7th Edition)” and amended by the International Nephropathy Association. The TCM symptom patterns of patients with HSPN were diagnosed according to the diagnostic standards amended by the Nephropathy Committee of Chinese Medical Ideology, the Kidney Disease Committee of Integrated Traditional Chinese and Western Medicine, and our clinical experiences. The diagnostic standards of TCM syndromes are formulated according to books “Chinese Traditional Medicine New Drug Clinical Research Guiding Principle,” “Clinical Guideline of New Drugs for Traditional Chinese Medicine,” “Pediatrics of Chinese Medicine,” and “Diagnostics of Traditional Chinese Medicine.” Every TCM syndrome includes clinic expression, main symptoms, and secondary symptoms [9–11].

### 2.3. TCM Symptom Patterns of Patients with Henoch-Schönlein Purpura Nephritis (HSPN)

(1) “Dampness-heat accumulation” includes the following clinical symptoms and signs:

*Main Symptoms.* Red or amaranth rash often occurs in batches; rashes often merge into pieces, even blood blister. The spots are seen on the lower limbs and buttocks or around the joints: skin furuncle, swelling and sore throat, dry mouth but not thirst, abdominal distention and pain, reduced appetite, brown urine, burning sensation during urination, difficulty and pain in micturition, loose stools,and tongue with yellow greasy coating.

*Secondary Symptoms. *Heavy body, weakness of limbs, bitter or dry mouth, mouth stick, nausea vomiting, jaundice (yellow eyes and yellow skin), edema of the face and limbs, and frequent pulse or slippery pulse.

(2) “liver-kidney yin deficiency” includes the following clinical symptoms and signs:

*Main Symptoms. *Bright red rash, dysphoria (fever) in chest, palms, and soles, dry throat, heatiness, lumbar debility, red tongue, and less fur.

*Secondary Symptoms. *Spermatorrhea, semen leakage or menstrual disorder, dry eyes or blurred vision, giddy tinnitus, and pulse thin or counting.

(3) “Qi and yin deficiency” includes the following clinical symptoms and signs:

*Main Symptoms. *Rash faint, lubricious complexion, shortness of breath, lassitude or easy cold, afternoon or evening low-grade fever, feverishness in palms or soles, and lumbago or edema.

*Secondary Symptoms. *Dry throat, heatiness, pharyngeal dull-red, pharyngodynia, red or reddish tongue, less tongue fur, and thin or weak pulse.

(4) “Purpura with syndrome of blood-heat bleeding” includes the following clinical symptoms and signs:

*Main Symptoms.* There are lots of bright red or amaranth skin petechiae and ecchymoses; the rashes were widely distributed and even fused into pieces. The rashes changed rapidly and appeared in batches: thirst to drink, vexation, and agitation, red or fuchsia tongue with burr, and tongue with thin or heavy yellow greasy coating.

*Secondary Symptoms. *Red face or red lip, halitosis, sore gums or bleeding, constipation, and powerful pulse.

### 2.4. Diagnostic Criteria

Every syndrome includes 3 items of main symptoms (tongue examination is necessary) or 2 items of main symptoms + 2 items of secondary symptoms.

### 2.5. Cases of Exclusion Criteria

(1) The first case is renal damage caused by diseases such as systemic lupus erythematosus (SLE) and viral hepatitis (hepatitis b, hepatitis c, etc.)

(2) The second case is hematuresis caused by high urinary calcium and left renal vein compression syndrome.

### 2.6. Statistical Analysis

SPSS version 23.0 software (SPSS Inc., Chicago, IL, USA) was used to analyze data. Quantitative data are expressed as the mean ± standard deviation (mean ± SD). Differences in measurement data were compared using the *χ*^2^ test. *t*-test was used for quantitative data conforming to a normal distribution, and rank test was used for quantitative data not conforming to a normal distribution. *P* values < 0.05 were considered to denote significant differences.

## 3. Results

### 3.1. General Clinical Conditions

In 262 children of HSPN, the ratio of male/female is 1.09 : 1, with an average age of 10.07. There are no obvious differences in age, sex, etiology, and pathogenesis; the causes of the disease are complicated; some are not very clear at present and some other causes are less correlated for the disease; allergy infection is the important triggering factor. Acute nephritis, the accelerated nephritis, and chronic nephritis are not included in the study.

### 3.2. Analysis of Distribution of Patients

As shown in Table 1, in 262 children of HSPN, dampness-heat accumulation made the dominant rate of 59.9%, deficiency of liver-yin and kidney-yin accounted for 17.6%, qi and yin deficiency ratio reached 12.6%, and blood-heat bleeding accounted for 9.9%.

### 3.3. Analysis of 24 h Urinary Protein

Table 2 indicated that hematuria (24 h urinary protein) was heavier in dampness-heat accumulation internal patients; it showed statistical difference among the deficiency of liver-yin and kidney-yin symptoms (*P* = 0.043) and qi and yin deficiency symptoms (*P* = 0.026).

### 3.4. Analysis of Hematuresis

As shown in Table 3, moderate or severe hematuresis usually happens to dampness-heat accumulation pattern in HSPN patients, accounting for 58.0%. Mild hematuresis usually happens to liver-kidney yin deficiency and qi and yin deficiency patterns in HSPN patients, accounting for 73.3% and 51.5%. There was a significant statistical difference between the dampness-heat accumulation pattern and qi and yin deficiency pattern (*P* < 0.001).

### 3.5. Analysis of Immune Globulin

As shown in Table 4, IgG of the patients in different TCM patterns of symptoms were within normal values. IgM of the patients in dampness-heat accumulation and deficiency of both qi and yin pattern was higher than normal values; there was no statistical difference with IgM and IgG among the different TCM patterns of symptoms (*P* > 0.05); IgA in all patterns was higher than the normal range; there was no statistical difference between the different TCM patterns of symptoms (*P* > 0.05). IgE in all patterns was higher than the normal range; dampness-heat accumulation pattern was much higher than the other groups; there was no statistical difference between the different TCM patterns of symptoms (*P* > 0.05).

### 3.6. Analysis of T Lymphocytes

As shown in Table 5, in qi and yin deficiency pattern, the levels of CD4 cells and the CD4/CD8 ratio are lower than normal range, while CD8 cells are higher than normal range. The levels of CD4 cells, CD8 cells, and CD4 / CD8 ratio were statistically significant in Qi and yin deficiency pattern and dampness-heat accumulation pattern (*P* = 0.006, 0.032, and 0.001); CD3 cells are all in the normal range among four different TCM patterns of symptoms, showing no statistical difference.

### 3.7. Analysis of Blood-Coagulating Indexes

As shown in Table 6, D-D values in in dampness-heat accumulation and blood-heat bleeding pattern are more than 4 times higher than the normal reference value; D-D value in liver-kidney yin deficiency is slightly higher than the normal reference value; the dampness-heat accumulation pattern shows significant statistical difference among qi and yin deficiency pattern and liver-kidney yin deficiency pattern (*P* = 0.017 and *P* = 0.036); the values of PT, FBI, and APTT in all patterns are approximately in normal range; there was no statistical difference among all patterns (*P* > 0.05).

## 4. Discussion

Some researches showed that HSPN develops in 18–81% of Henoch-Schönlein purpura nephritis patients, and the long-term outcomes of this nephritis show great variation. A nephrotic state at disease onset has been proposed as a predictor of poor renal outcomes [12–15].

Therefore, detailed investigations and studies of the pathogenesis on children with HSPN are still necessary. In this article, we decided to review the status of the research on the HSPN disease from the perspective of Chinese and Western medicine.

Although the name of disease “purpura” never appeared in the ancient books of traditional Chinese medicine, its symptoms can be found in the description of various diseases. Symptoms for rashes can be classified as “spot,” “spontaneous bleeding of the flesh,” and “grape epidemic” symptoms of disease; symptoms for hematuresis were attributed to the category of diseases “blood trouble” and “hematuria”; symptoms for edema (nephrotic syndrome) can be classified to the diseases of “edema” [16]. With the development of combination of Chinese and western medicine, many Chinese scholars have standardized the disease according to the name of allergic purpura nephritis.

TCM theory made the point that the etiology and pathogenesis of the HSPN included three important factors: natural endowment, invasion of exogenous evil, and pathological products. The illness aggravated or relapsed based on a variety of pathological products (wet, poison, blood stasis, deficiency, etc.). Asthenia of both the spleen and kidney is the pathogenic basis of HSPN. (1) Natural endowment: TCM ancient books “Miraculous Pivot” and “General Treatise on the Cause and Symptoms of Diseases” indicated that children are deficient in qi and blood and are susceptible to virus infection. Furthermore, L. Liu and F. J. Liu [15] summarized that unique feature in children with deficiency in qi and blood of the body is the immanent reason that led to children's Henoch-Schönlein purpura. Previous studies identified that, in HSPN patients, predisposing risk factors like bacteria attached to the dust, seafood, animal fur, the cold air, pollen, and some drugs that contain antibiotics and so forth [11–13]. Allergic constitution could be also considered as an abnormal natural endowment, which plays a dominant role in the pathogenesis of HSPN. (2) Invasion of exogenous evil: yun-qi theory (“Huangdi's Internal Classic”) studied nature's change regulation and emphasized the impact of climate change on biology, especially on human beings. Most TCM theories illuminated that wind heat and damp heat evil invading the body is the most common exogenous pathogenic factor in allergic purpura nephritis. HSPN often occurred in the winter and spring and has obvious seasonality. When the climate is warm or windy, exogenous evil can easily invade body. (3) Pathological products: according to TCM theory, the occurrence of diseases in coordination between yin and yang and the treatment of diseases is the reestablishment of the equilibrium between them. Yin deficiency can led to yin-yang disharmony; due to the imbalance of yin and yang after yin deficiency, yang heat excessed, which can also help the heat of evil. Therefore, dampness heat and yin deficiency affect each other's vicious cycle. Chronic disease led to kidney deficiency and blood stasis [17]. The vicious cycle begins, thus inducing the results shown in Table 2. In 262 children of HSPN, dampness-heat accumulation made the dominant make-up of the TCM patterns of syndrome. Moreover, Tables 2 and 3 indicated that hematuria and proteinuria were heavier in dampness-heat accumulation internal patients.

In the theory of TCM, the etiology and pathogenesis of HSPN summarized the contents of wind, heat, humidity, poison, blood stasis, and deficiency syndrome. The viscera and meridians in children are tender and delicate; vital energy flow and spirit are not fully filled in children's body. The body could not effectively resist external environment and was susceptible to invasion of external pathogens, resulting in accumulation of dampness heat and liver-qi depression. In addition to improper diet or disturbance of the zang-fu functions, it easily caused damp-heat or noxious blood stasis, exopathogen or internal evil damage, and blood-collateral that induced the disease. This paper discusses the relationship between renal hematuria and kidney collateral based on collateral disease theory. The main pathogenesis may be phlegm dampness and blood stasis. In the case of dampness heat invading the body, the accumulation of toxin results in the formation of proteinuria. The illness in children is being aggravated and became incurable disease. Some studies indicated that the recipe for removing dampness, promoting diuresis, nourishing kidney, and consolidating essence is an effective therapy that can improve the body's immune function and then alleviate the internal hypercoagulable state which contribute to the recovery of allergic purpura nephritis [16, 18].

HSPN, also called renal allergic purpura, is a systemic syndrome characterized by small vessel damage. It is characterized by skin purpura, renal damage, hemorrhagic gastroenteritis, arthritis, and so forth [2]. The cause of the disease is not clear, and it may be associated with infection and allergy. Some cases are infected before the onset of the disease; the most common is the upper respiratory tract infection. Many patients have food or drug allergies [3]. At present, HSPN is considered as an immune complex disease, and IgA plays an important role in the disease [4]. Other immune abnormalities also regulate cell changes by T lymphocyte, anaphylaxis mediated by IgE, and so firth [19]. The disease usually occurs more than 1~3 days after the upper respiratory tract infection or other inflammatory reactions. Studies have shown that IgA nephropathy has immune dysfunction, accompanied by immune response and immune complex deposition. Cytokines and inflammatory factors may lead to glomerular mesangial hyperplasia and glomerulosclerosis. Inadequate inhibition of inflammatory cells leads to abnormal activation of lymphocytes, immune function, and renal vascular wall forming immune complex IgA, leading to vasculitis changes.

T lymphocytes include CD8 cells and CD4 cells; they play a very complex role during the process of immune function and possess different activity in different period. Most of them can be induced or inhibited by themselves or others. Under normal condition, the CD4/CD8 ratio maintains dynamic balance and keeps the body's immune function stable. When the ratio is higher than normal, the immune state is hyperactivity, and the ratio decreased, or even inversion indicates that the immune state was very poor [19–21].

As shown in Tables 4 and 5, IgE in all patterns is higher than the normal range; dampness-heat accumulation is significantly higher than the other groups. Some researches show that IgE is closely related to allergic reactions. The increases of IgE induced an allergic reaction in children with HSPN [22–24]. In qi and yin deficiency pattern, the levels of CD4 cells and the CD4/CD8 ratio are lower than normal range. Previous studies confirmed that immunological dysfunction in children with HSPN manifests as reduced levels of CD3 cells and CD4 cells and also as lowered immunological function of cells. These changes lead to immune deficiency and stimulate the secretion of inflammatory factors. The IgA immune complex is deposited on the capillary wall of glomeruli [25, 26]. Some researches indicated that the HSPN patients have a low cell immune function and an imbalanced T cell subclone, polyclonal B cells activation, and immunoglobulin secretion abnormalities which may be related to the development of HSPN [27].

The low function levels of Th1 and TS cells existed in patients diagnosed with HSPN. The mechanism of the disease may be due to polyclonal B cells activation and abnormal immunoglobulin secretion. IgA immune complex deposits in the glomerular capillary wall, causing glomerular capillary inflammation and thrombosis, leading to kidney damage [2, 3].

The immune mechanism in HSPN indicated that the treatment may be according to regulated immune function. On one hand, it can reduce the deposition of immune complexes in the glomeruli, thereby reducing the immune and inflammatory response, improving the pathological damage of kidneys. On the other hand, it can decrease the chance of infection and thus facilitate the recovery of kidney disease.

Previous studies confirmed that obstacles in the glomerular capillary blood coagulation and fibrinolysis are one of the decisive factors in forming glomerulonephritis irreversible lesions, which can lead to irreversible transformation in kidney tissue damage and renal insufficiency [28–30]. Anticoagulant therapy can enhance the speed of glomerular repair, improve renal blood circulation, and promote renal lesion repair and fibrin absorption, which can improve the degree of renal pathological damage [31, 32]. Previous study revealed that the therapy for clearing away heat, promoting diuresis, nourishing the kidney, and consolidating essence is effective in children with HSPN from internal accumulation of damp-toxin [18]. Some researches considered that higher levels of D-D are an important marker of hypercoagulability and hyperfibrinolysis. The higher the level of FIB and D-D, the earlier the kidney damage and the worse the prognosis of the diseases [33]. It is indicated that coagulation mechanism may also play an important role in the pathogenesis of this disease. Table 6 in this article tested and verified the point of view.

## 5. Conclusion

In summary, this study demonstrates that dampness-heat accumulation syndrome was dominant in HSPN patients and clinical symptoms like hematuria and proteinuria were heavier in the dampness-heat accumulation patients. These clinical symptoms were always accompanied by immune disorders and coagulation disorders. This helps us understand the clinical significance of TCM symptom patterns in the diseases studies and thus provide the most curative effects of Chinese herbs decoction for clinical treatment.

## Figures and Tables

**Table 1:** The distribution of TCM patterns of symptoms in 262 HSPN patients.

Pattern	Cases	Constituent ratio (%)
Dampness-heat accumulation	157	59.9
Liver-kidney yin deficiency	45	17.6
Qi and yin deficiency	33	12.6
Blood-heat bleeding	24	9.9
Total	262	100

*Notes*. TCM: traditional Chinese medicine; HSPN: Henoch-Schönlein purpura nephritis.

**Table 2:** Mean concentrations of 24 h urinary protein in 262 HSPN patients with different TCM patterns of symptoms (mean ± SD).

Pattern	Cases	24 h urinary protein (mg/kg/d)
Dampness-heat accumulation	157	34.16 ± 4.23
Liver-kidney yin deficiency	45	11.78 ± 3.60
Qi and yin deficiency	33	12.25 ± 4.08
Blood-heat bleeding	24	18.91 ± 3.75

*Notes*. TCM: traditional Chinese medicine; HSPN: Henoch-Schönlein purpura nephritis.

**Table 3:** Mean concentrations of hematuresis in 262 HSPN patients with different TCM patterns of symptoms.

Pattern	Cases	RBC 0–2/HP (normal)	RBC 3–5/HP (mild)	RBC 6–20/HP (moderate)	RBC >20/HP (severe)	Mild + moderate	Moderate + severe
Dampness-heat accumulation	157	15 (9.5%)	51 (32.5%)	68 (43.3%)	23 (14.7%)	119 (75.8%)	91 (58.0%)
Liver-kidney yin deficiency	45	3 (6.7%)	33 (73.3%)	9 (20.0%)	0 (0)	42 (93.3%)	9 (20.0%)
Qi and yin deficiency	33	9 (27.3%)	17 (51.5%)	7 (24.2%)	0 (0)	25 (75.7%)	8 (24.2%)
Blood-heat bleeding	24	1 (4.2%)	13 (54.1%)	9 (37.5%)	1 (4.2%)	7 (91.6%)	10 (41.7%)

*Notes*. TCM: traditional Chinese medicine; HSPN: Henoch-Schönlein purpura nephritis; RBC: red blood cell. Diagnostic criteria in hematuresis: normal range: RBC 0–2/HP; mild range: RBC 3–5/HP; moderate range: RBC 6–20/HP; severe range: RBC > 20/HP.

**Table 4:** Mean quantitative value of immune globulin in HSPN patients with different TCM patterns of symptoms (mean ± SD).

Pattern	IgA (g/L)	IgG (g/L)	IgM (g/L)	IgE (IU/mL)
Dampness-heat accumulation	2.93 ± 0.26^*∗*^	7.53 ± 0.81	1.54 ± 0.32^*∗*^	259.12 ± 21.03^*∗*^
Liver-kidney yin deficiency	2.75 ± 0.44^*∗*^	7.51 ± 1.21	1.43 ± 0.29	105.98 ± 10.14^*∗*^
Qi and yin deficiency	2.63 ± 0.23^*∗*^	8.01 ± 1.35	1.68 ± 0.49^*∗*^	124.73 ± 15.21^*∗*^
Blood-heat bleeding	3.02 ± 0.38^*∗*^	8.94 ± 0.94	1.09 ± 0.38	119.64 ± 11.23^*∗*^

*Notes*. TCM: traditional Chinese medicine; HSPN: Henoch-Schönlein purpura nephritis; Ig: immune globulin, IgA: immunoglobulin A; IgM: immunoglobulin M; IgG: immunoglobulin G; IgE: immunoglobulin E. *∗* shows higher than normal reference value. Range of normal value in immune globulin: IgA: 0.7–2.3 g/L; IgG: 3–10 g/L; IgM: 0.4–1.5 g/L; IgE: ≤87 IU/ml.

**Table 5:** Mean quantitative value of T lymphocytes in 262 HSPN patients with different TCM patterns of symptoms (mean ± SD).

Pattern	CD3 (%)	CD4 (%)	CD8 (%)	CD4/CD8 (%)
Dampness-heat accumulation	68.02 ± 5.01	36.15 ± 3.68	29.00 ± 3.74	1.25 ± 0.38
Liver-kidney yin deficiency	69.30 ± 5.03	30.25 ± 4.05	34.21 ± 4.34	0.88 ± 0.21^△^
Qi and yin deficiency	62.18 ± 5.46	23.73 ± 4.15^△^	36.75 ± 3.58^*∗*^	0.65 ± 0.19^△^
Blood-heat bleeding	63.45 ± 4.32	34.18 ± 3.58	30.14 ± 4.63	1.13 ± 0.30

*Notes*. TCM: traditional Chinese medicine; HSPN: Henoch-Schönlein purpura nephritis; T cell: T lymphocytes. *∗* shows higher than normal reference value; △ shows lower than normal reference value. Range of normal value in CD series: CD3: 55–82%; CD4: 25–57%; CD8: 9–35%; CD4/CD8: 1.1–2.

**Table 6:** Mean quantitative value of blood-coagulating indexes in 262 HSPN patients with different TCM patterns of symptoms (mean ± SD).

Pattern	PT (s)	APTT (s)	FIB (g/L)	D-D (ug/ml)
Dampness-heat accumulation	12.25 ± 1.08	39.38 ± 6.33	3.65 ± 1.08	2.30 ± 0.46^*∗∗*^
Liver-kidney yin deficiency	14.34 ± 0.56	40.81 ± 4.75	2.98 ± 0.35	0.65 ± 0.13^*∗*^
Qi and yin deficiency	12.58 ± 0.64	42.13 ± 5.05	3.41 ± 0.85	0.53 ± 0.27^*∗*^
Blood-heat bleeding	13.17 ± 0.78	39.13 ± 1.96	3.56 ± 0.48	2.28 ± 0.76^*∗∗*^

*Note*. TCM: traditional Chinese medicine; HSPN: Henoch-Schönlein purpura nephritis; APTT: activated partial thromboplastin time; D-D: D-dimer; PT: prothrombin time; FIB: fibrinolysis. *∗* shows less than 4 times higher than normal reference value; *∗∗* shows more than 4 times higher than the normal reference value. Range of normal value in coagulation function indexes: PT: 11–15 s; APTT: 28–45 s; FIB: 2–4 g/L; D-D: 0.01–0.5 ug/ml.

## References

[1] Kawasaki Y. (2011). Mechanism of onset and exacerbation of chronic glomerulonephritis and its treatment. *Pediatrics International*.

[2] Kawasaki Y. (2011). The pathogenesis and treatment of pediatric Henoch-Schönlein purpura nephritis. *Clinical and Experimental Nephrology*.

[3] Lau K. K., Suzuki H., Novak J., Wyatt R. J. (2010). Pathogenesis of Henoch-Schönlein purpura nephritis. *Pediatric Nephrology*.

[4] Kiryluk K., Moldoveanu Z., Sanders J. T. (2011). Aberrant glycosylation of IgA1 is inherited in both pediatric IgA nephropathy and Henoch-Schönlein purpura nephritis. *Kidney International*.

[5] Kanai H., Sawanobori E., Kobayashi A., Matsushita K., Sugita K., Higashida K. (2011). Early treatment with methylprednisolone pulse therapy combined with tonsillectomy for heavy proteinuric henoch-schönlein purpura nephritis in children. *Nephron Extra*.

[6] Liu Y., Xu H. Y. (2013). Abnormal IgA glycosylation in children with henoch-schonlein purpuric nephritis. *Practical Clinical Medicine*.

[7] Kang H., Zhao Y., Li C. (2015). Integrating clinical indexes into four-diagnostic information contributes to the Traditional Chinese Medicine (TCM) syndrome diagnosis of chronic hepatitis B. *Scientific Reports*.

[8] Teng L., Zhang J., Dai M., Wang F., Yang H. (2015). Correlation between Traditional Chinese Medicine symptom patterns and serum concentration of zinc, iron, copper and magnesium in patients with hepatitis B and associated liver cirrhosis. *Journal of Traditional Chinese Medicine*.

[9] Ren X. Z., Li W. (2013). Clinical study on TCM Syndromes of children with Henoch Schonlein nephritis. *Zhong Hua Zhong Yi Yao Za Zhi*.

[10] Hu Y., Yao Y., Liu J. (2009). PeiXueYi veteran doctor of traditional Chinese medicine experience in the treatment of allergic purpura. *Journal of Emergency in Traditional Chinese Medicine*.

[11] Ren Z X., Wei LI. (2013). Clinical study on the regulation of TCM syndromes with purpura nephritis in children. *China Journal of Traditional Chinese Medicine & Pharmacy*.

[12] Wakaki H., Ishikura K., Hataya H. (2011). Henoch-Schönlein purpura nephritis with nephrotic state in children: Predictors of poor outcomes. *Pediatric Nephrology*.

[13] Baltag M. A., Brumariu O., Munteanu M., Mihaila D. (2010). Henoch-Schonlein nephritis: diagnosis and prognosis problems in childhood. *Revista Medico-Chirurgicală a Societăţii de Medici şį Naturalişţi din Iaşį*.

[14] Edström Halling S., Söderberg M. P., Berg U. B. (2010). Predictors of outcome in Henoch-Schönlein nephritis. *Pediatric Nephrology*.

[15] Liu L., Liu F. J. (2015). Clinical and pathological analysis in children with Henoch-Schonlein purpura nephritis. *Journal of Clinical Pediatrics*.

[16] Chen F., Zhen X. F., Yan H. M. (2011). Therapy for promoting dieresis and removing toxin used to treat 30 child patients with Henoch-Schonlein Purpuric Nephritis. *Zhong Guo Zhong Yi Ji Zheng*.

[17] Chen S. M., MO Y. (2001). Aetiological factor and pathogenesis of Henoch-Schonlein purpura nephritis. *Chinese Journal of Practical Pediatrics*.

[18] Ding D., Yan H., Zhen X. (2014). Effects of Chinese herbs in children with Henoch-Schonlein purpura nephritis: a randomized controlled trial. *Journal of Traditional Chinese Medicine*.

[19] Chen D. Y., Zheng M., Huang X. W. (2012). The correlation of serum fibrinogen inhibition C and T lymphocyte subsets in Children allergic purpura. *Guangdong Medical Journal*.

[20] Wu Y. Y. (2013). The changes and significance of immune globulin and T lymphocyte subsets in children acute allergic purpura. *Jiangsu Medical Journal*.

[21] Zhun Y. Q., Wang Q. L., Wu S. Y. (2013). Clinical characteristics and changes of blood lymphocytes in children with HSP. *Zhe Jiang Yi Xue*.

[22] Ding Y., Yin W., He X. L., Xiong Y. H., Peng F. (2013). Exploration of immune function at acute phase in children with Henoch-Schonlein purpura. *Zhong Guo Mian Yi Xue Za Zhi*.

[23] (2016). Effect of tripterygium glycosides and Danshen injection on blood coagulation mechanism in children with allergic purpura nephritis. *China Journal of Chinese Materia Medica*.

[24] Wang W. L., Wang J. G. (2012). Clinical significance of serum total IgE and eosinophil cationic protein in allergic purpura nephritis infants. *China Modern Doctor*.

[25] Chen X. H., He F., Liu Q. (2013). The determination and significance of serum IL-5, IL-10, CD+4 CD+25 regulatory T cells in kidney type allergic purpura patients. *Chinese Journal of Difficult and Complicated Cases*.

[26] Bi Y. N., Wang L. Z., Xi X. Q. (2013). The significance of level change with peripheral blood CD+4-CD+ (28) and CD+8-CD+ (28) -T cells of incipient allergic purpura in children. *Shan Dong Yi Yao*.

[27] Chen J., Song X. X., Wang L. F. (2009). Correlations of immunopathologic types with clinical manifestations and histological classifications in children with henoch-schonlein purpura nephritis. *Joural of Applied Clinical Pediatrics*.

[28] Fu H. M., Ni J. X., Li P. (2004). The changes of Von-willbrand factor, D-Dimer and APTT, PT levels in patients with henoch-schonlein purpura. *Joural of Applied Clinical Pediatrics*.

[29] Wang Z. J. (2012). The Value of Early Judgement by Detection of FIB, FDP and D-D in Children with henoch-schonlein purpura caused renal damage. *Journal of Qinghai Medical College*.

[30] Liu Y. P., Chen D. Y., Huang X. W. (2012). Value of D-dimer, Cystatin C and Immunoglobulin in Childhood Henoch-Schonlein Purpura. *Chinese General Practice*.

[31] Liu Y. Q., Ding Y. (2012). Experiences of Ding Ying in treating allergic purpura nephritis. *Journal of Traditional Chinese Medicine*.

[32] Hou L. Y., Yan H. M., Zhao Q. (2012). Clinical observation of 40 cases children allergic purpura nephropathy Combining traditional Chinese and western medicine treatment. *Journal of Emergency in Traditional Chinese Medicine*.

[33] Ma Y., Lu L., Deng F. (2011). Changes of plasma fibrinogen, D-dimer, fibrin degrada tion products associated with kidney injury in children with Henoch-Schonlein purpura nephritis. *Acta Universitatis Medicinalis Anhui*.

